# Results of Combined Penetrating Keratoplasty and Pars Plana Vitrectomy Performed for Infectious Keratitis with Endophthalmitis Compared to Other Non-Infectious Indications: Series of 129 Eyes

**DOI:** 10.3390/jcm14196748

**Published:** 2025-09-24

**Authors:** Shady Suffo, Loay Daas, Alaa Din Abdin, Ibrahim Qozat, Cristian Munteanu, Berthold Seitz, Yaser Abu Dail

**Affiliations:** Department of Ophthalmology, Saarland University Medical Center, 66421 Homburg, Germany; shady.suffo@uks.eu (S.S.); loay.daas@uks.eu (L.D.); alaadin.abdin@uks.eu (A.D.A.); ibrahimqozat@usf.edu (I.Q.); cristian.munteanu@uks.eu (C.M.); berthold.seitz@uks.eu (B.S.)

**Keywords:** combined vitreoretinal surgery, infectious keratitis, endophthalmitis, penetrating keratoplasty, traumatic ocular injury

## Abstract

**Background/Objectives:** The aim of this study was to determine the frequency of the indications and compare the results and prognosis of combined penetrating keratoplasty (PKP) and vitreoretinal surgery (PKPVR) performed for infectious keratitis with endophthalmitis (IKE) to those performed for other non-infectious indications in a German university eye hospital. **Methods:** Medical records were searched for patients who underwent PKPVR between 2016 and 2024. Demographic data, indication, best corrected visual acuity (BCVA), and intraocular pressure (IOP) at the first and last visits; data on conservative and surgical treatment; and data on the development of phthisis bulbi and the need for enucleation were recorded. **Results:** A total of 129 eyes of 128 patients were included in this retrospective study (61 ± 22 years, male: 64%). Of these eyes, 50% were treated for IKE and 50% for other non-infectious indications. The mean follow-up time was 24 ± 23 months, BCVA improved from logMAR 2.3 ± 0.5 to 2.0 ± 0.7 at the last visit (*p* < 0.01), and the percentage of severe visual impairment (logMAR ≥ 1.3) decreased postoperatively from 97% to 86%. A total of 9/129 eyes were eventually enucleated (7%), and another 5/129 had developed phthisis bulbi at the last visit (4%). Compared to the non-infectious group, the IKE-group had a significantly higher enucleation rate (*p* = 0.05) and also a higher rate of significant visual improvement (from logMAR ≥ 1.3 to <1.3) (*p* = 0.04). Eyes which achieved a significant BCVA improvement in the IKE-group had a significantly lower rate of retinal infiltration, hemorrhage, and ischemia (*p* = 0.03). **Conclusions:** PKPVR is an indispensable procedure for eliminating infection in eyes with IKE. Compared to other non-infectious indications, the IKE-group had the highest rate of both enucleation and significant BCVA improvement.

## 1. Introduction

Combined penetrating keratoplasty (PKP) and vitreoretinal surgery (PKPVR) is a surgical procedure used in patients who require simultaneous PKP and pars plana vitrectomy (PPV) due to posterior segment morbidity and significant corneal opacities such as corneal infiltration, scarring, or edema [1,2]. Indications for PKPVR include infectious keratitis with endophthalmitis (IKE) and other non-infectious indications such as severe traumatic ocular injuries and retinal detachment and tractive membranes with concurrent corneal opacity (video 1: https://www.youtube.com/watch?v=GQVsxuC2Gys, accessed on 30 July 2025). Treatment options in these cases include PPV with a temporary keratoprosthesis (TKP), open-sky vitrectomy, endoscopic PPV, and PKP directly followed by PPV [3].

Endophthalmitis is a medical emergency in which the vitreous is affected by a fungal or bacterial infection. It could occur after ocular surgery or penetrating ocular trauma or as a consequence of severe corneal infection [4]. If not diagnosed and treated properly, it can lead to severe visual loss or even the loss of the eye [5,6]. In patients with concomitant corneal infection and endophthalmitis, PKPVR is indispensable to preserve visual function and the integrity of the eye.

Mechanic ocular injuries are also medical emergencies and major causes of blindness [7]. Traumatic eye injuries range from simple corneal or conjunctival erosions to severe simultaneous corneal, scleral, lenticular, and retinal injuries, as well as intraocular hemorrhage [8]. Severe injuries may also require PKPVR to preserve eye function and integrity.

The aim of this study is to determine the frequency of the indications of PKPVR in a large cohort of 128 patients and 129 eyes in a German university eye hospital and to compare the results and prognosis of PKPVR between patients with IKE along with other non-infectious indications.

## 2. Patients and Methods

This is a retrospective cohort study to determine the frequency of various indications of PKPVR, including IKE and other non-infectious indications such as traumatic ocular injury, retinal detachment with severe corneal opacity, and IOL luxation into the vitreous and tractive retinal membranes (epiretinal membrane and/or proliferative vitreoretinal membrane) with severe corneal opacity, and to compare their prognosis with regard to pre- and postoperative visual acuity and the development of phthisis bulbi and/or enucleation within a minimum follow-up time of 3 months. This study was conducted in accordance with the Declaration of Helsinki and approved by the Ethics Committee of the Medical Association of Saarland, Germany (Nr.50/22). Written informed consent was not collected due to the retrospective character of this study. All data were gathered as part of the regular clinical examination and therapy process.

The medical files of patients who underwent PKPVR between 2016 and 2024 at the Department of Ophthalmology, Saarland University Medical Centre, Saarland, 5Germany, were reviewed.

The collected data included demographic data; indication of PKPVR; previous and consequent ocular surgeries, including PKP, PPV, surgical treatment for glaucoma, and amniotic membrane transplantation (AMT); pre- and postoperative best corrected visual acuity (BCVA); follow-up time; intraoperative retinal findings (retinal infiltration, bleeding, and/or ischemia); development of phthisis bulbi; need for enucleation; and anatomical success criteria including retinal attachment and corneal clarity, as well as intraocular pressure (IOP) at the last visit. BCVA was converted to logMAR units to match the standard and to allow for a comparison with the results of other studies. The logMAR equivalent for counting fingers was considered 1.8, for hand motion 2.3, for light perception 2.7, and for nulla lux 3 [9,10]. In alignment with the definition of severe visual impairment in Germany [11], significant visual improvement was defined as an improvement in BCVA from ≥1.3 logMAR preoperatively to BCVA < 1.3 postoperatively. Anatomical success was considered achieved if the eye showed a clear corneal graft, retinal attachment, and normal IOP at the last visit [3,12]. Patients with less than 3 months of follow-up time were excluded from the longitudinal analysis of prognosis assessment.

Data from 129 eyes of 128 patients were analyzed. We divided the patients into an IKE-group (65 eyes) and non-infectious group (64 eyes). The IKE-group was then divided into two subgroups based on the improvement in BCVA in patients with a follow-up time of at least 3 months: a significant BCVA improvement group (BCVA improvement from ≥1.3 logMAR preoperatively to <1.3 logMAR postoperatively, 9 eyes) and non-significant BCVA improvement group (BCVA ≥ 1.3 logMAR both preoperatively and postoperatively, 41 eyes). All eyes in the IKE-group with a follow-up time of at least 3 months had a BCVA ≥ 1.3 logMAR at the baseline visit. The non-infectious group was also further divided into 4 subgroups according to indication as previously mentioned. Patients were only included if they received PKP and PPV simultaneously; patients who underwent open-sky vitrectomy accompanied with PKP were excluded.

PKPVR was performed under general anesthesia with Atracurium as a muscle relaxant [13]. In eyes with a trephination diameter up to 8.0 mm, an Eckardt temporary keratoprosthesis (TKP) [14] was used to ensure the good visualization of the posterior ocular segment during PPV, and the transplantation was completed afterwards through fixing the donor cornea to the recipient’s bed using 24 to 32 interrupted sutures. In eyes where a trephination diameter > 8 mm was necessary, PKP was first completed and then followed by PPV in the same operation to restore the ocular globe integrity and reduce the risk of subchoroidal expulsive hemorrhage. In aphakic eyes and in eyes in which an IOL extraction was also simultaneously performed, a Flieringa ring was used to prevent the collapse of the eyeball during the procedure. No open-sky vitrectomy was performed in our department between 2016 and 2024.

Statistical analysis was performed using the Excel program (Microsoft). Continuous data were described as the mean and standard deviation. Categorical variables were described as percentages. If continuous variables were normally distributed, they were compared using Student’s *t*-test. Categorical variables were compared using the Chi-square test. A *p*-value of less than 0.05 was considered statistically significant.

## 3. Results

A total of 129 eyes of 128 patients (age: 61 ± 22 years) were included in this study. The mean follow-up time was 24 ± 23 months. Demographic data are summarized in Table 1. A total of 65/129 (50%) of the eyes were treated for IKE, 25/129 (20%) were treated after ocular trauma, 16/129 (12%) were treated for retinal detachment, 16/129 (12%) were treated for epiretinal membrane, and 7/129 (6%) were treated for IOL luxation in the vitreous. Moreover, 9/50 eyes with IKE showed significant BCVA improvement at the last visit, while 41/50 eyes showed non-significant BCVA improvement at the last visit. The IKE-group was significantly older (68 ± 22 years) than the non-infectious group (54 ± 22 years) (*p* < 0.001). The number of male patients was significantly higher in the non-infectious group (77%) compared to the IKE-group (52%) (*p* = 0.04). A total of 24 eyes in the IKE-group had a bacterial infection, 8 had a mycotic infection, and 5 had a bacterial and mycotic co-infection. The etiology was otherwise unknown in 28 eyes.

The surgical medical history of the eyes is summarized in Table 1. Patients in the non-infectious group had significantly more PPV (44%) and significantly fewer AMT (20%) procedures before PKPVR compared to the IKE-group (PPV 18%, AMT 49%) (*p*-value 0.02 and <0.001, respectively). There was no significant difference in PKP and glaucoma surgical treatment between the two groups preoperatively.

A total of 107 eyes had a follow-up time of at least 3 months and were eligible for the longitudinal analysis. Patients with a follow-up time shorter than 3 months were significantly older compared to those with at least 3 months of follow-up time (75 ± 16 vs. 58 ± 22 years, *p* < 0.001). The mean follow-up time of this group was 24 ± 23 months. The results of BCVA, anatomical success, and postoperative surgical procedures are summarized in Table 2. The BCVA in the overall group was better at the last visit compared to the preoperative BCVA in 49% of the eyes, remained the same in 32% of the eyes, and got worse in 19% of the eyes. The number of eyes with severe visual impairment (BCVA ≥ 1.3 logMAR) was reduced from 97% preoperatively to 86% at the last visit. BCVA showed significant improvement in the overall group, as well as in the IKE-group, non-infectious group, and epiretinal membrane subgroup (*p* ≤ 0.02). The number of eyes which showed significant BCVA improvement was significantly higher in the IKE-group (19%) compared to the non-infectious group (6%) (*p* = 0.04).

**Figure 1:**
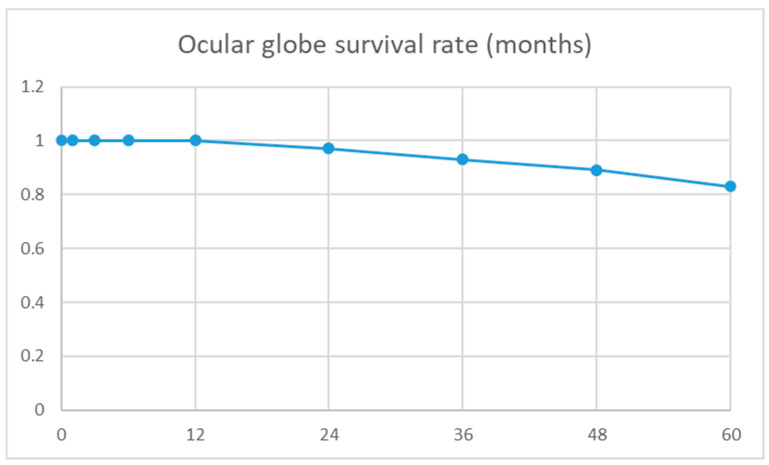
Ocular globe Kaplan–Meier survival rate, defined as absence of enucleation and/or phthisis bulbi.

A total of 9/129 (8.4%) eyes were enucleated, and 5/129 (4.6%) developed phthisis bulbi at the last visit. The number of eyes enucleated in the IKE-group was significantly higher (7/50) compared to that in the non-infectious group (2/57) (*p* = 0.05). The ocular globe survival rate, defined as the absence of enucleation and/or phthisis bulbi, was 97% at 2 years and 83% at 5 years (Figure 1). Moreover, 2/17 eyes with bacterial infection and none of the eyes with mycotic infection (0/7) were eventually enucleated (*p* = 0.3).

Anatomical success was achieved in 57% of the eyes in the overall group. The difference between the IKE-group (66%) and the non-infectious group (50%) was not statistically significant. Retinal attachment was achieved in 99% of the eyes, when excluding the eyes which underwent enucleation and/or with phthisis bulbi. A total of 13 eyes (14%) were, however, permanently filled with oil at the last visit. Overall, 66% of the eyes had a clear corneal graft at the last visit without a significant difference between the IKE-group and non-infectious group. Moreover, 83% of the eyes had a normal IOP at the last visit without a significant difference between the IKE-group and non-infectious group. The eyes permanently filled with oil at the last visit were significantly less likely to have a clear corneal graft (5/13, 38%) compared to the eyes without oil at the last visit (56/80, 70%) (*p* = 0.02).

Postoperatively, patients in the IKE-group underwent significantly more repeat PKP procedures (30% vs. 5%), significantly fewer glaucoma surgical procedures (6% vs. 26%), and significantly more AMT procedures (38% vs. 19%) compared to the non-infectious group (*p* ≤ 0.03). The number of repeat PPV and repeat PKPVR procedures was, however, statistically not significantly different.

The comparison between eyes with and without significant BCVA improvement in the IKE-group is summarized in Table 3.

The number of PPV and glaucoma surgical procedures preoperatively was lower in the group with significant BCVA improvement compared to the group with non-significant BCVA improvement (0% vs. 27% and 0% vs. 20%, respectively) (*p*-value 0.07 and 0.14, respectively). There was no significant difference between the two groups regarding the surgical postoperative procedures. Interestingly, the number of cases of intraoperative retinal bleeding, infiltration, and/or ischemia was significantly lower in eyes with significant BCVA improvement compared to the eyes with non-significant BCVA improvement (0% vs. 37%) (*p* = 0.03).

## 4. Discussion

Several studies have explored the results of PKPVR in patients with various indications. In the present cohort, 50% of PKPVR procedures were performed due to IKE. The late diagnosis and treatment of endophthalmitis can lead to visual loss and even increased mortality [15]. In eyes with exogenous endophthalmitis due to infectious keratitis, PKPVR is unavoidable and must be performed to eliminate the source of infection and prevent further ocular damage and the recurrence of the infection.

BCVA remained stable or improved postoperatively in 82% of the eyes in the IKE-group and in 81% of the eyes in the non-infectious group. BCVA also remained stable or improved in 83% of the eyes in the trauma subgroup in our cohort. This is similar to the findings of Bové Álvarez et al. [16], who reported a stable or improved BCVA in 86% of the eyes in the ocular trauma group and 78% of the eyes in the non-trauma group, and to the findings of Roters et al. [12] who reported a stable or improved BCVA at the last visit in 73% of the eyes. The rate was also better than the one reported by Zapata Cuevas et al. [17] (63%) in a case series of 32 eyes. Additionally, 17% of the eyes in our cohort achieved a BCVA ≤ 1.3 at the last visit, compared to 12% of the eyes in the cohort of Roters et al. [12], achieving 1 m vision or better at the last visit, and to 9% of the eyes in the case series of Cooney et al. [18]. In a case series of eyes with endophthalmitis which underwent PKPVR from Dave et al. [19], the rate of BCVA with logMAR ≤ 1.3 at the last visit was 25%, which is also similar to the rate in our IKE-group (22%).

The rate of phthisis bulbi was 5% in our cohort compared to 18% and 17% in the cohorts of Bové Álvarez et al. [16] and Roters et al. [12], respectively. It was also significantly lower than the reported rate in the endophthalmitis case series from Dave et al. [19] (21%). On the other hand, the rate of enucleation was somewhat higher in our cohort compared to Bové Álvarez et al. [16] and Roters et al. [12] (8% vs. 3% and 2%, respectively), likely due to the higher percentage of endophthalmitis in our cohort compared to that of Bové Álvarez et al. [16]. In a case series of 48 patients with endophthalmitis who underwent PKPVR, Velez-Montoya et al. [20] found an enucleation rate of 8.3%. The enucleation rate in the study of Dave et al. [19] with an endophthalmitis cohort was as high as 14%.

In the present study, patients in the IKE-group were significantly older compared to those in the non-infectious group. These results are in line with those of the studies of Bové Álvarez et al. [16] and Roters et al. [12] in which the patients in the trauma group were significantly younger (mean: 42 and 39 years, respectively) compared to those in the non-trauma group (mean: 56 and 56 years, respectively). This possibly reflects the higher frequency of neurotrophic keratitis in relatively older patients and the higher rate of traumatic ocular injuries in younger, working-age patients [21,22]. This could also explain the higher frequency of males in the non-infectious group compared to the endophthalmitis group and the higher rate of AMT pre- and postoperatively in the endophthalmitis group in this cohort. The findings hence emphasize the importance of the tight control and consequent treatment of patients with neurotrophic keratopathy and ocular surface defects in preventing such severe infectious complications. These findings are also in line with those of Velez-Montoya et al. [20], who reported the prevalence of corneal ulcers leading to a superinfection and consequent IKE requiring PKPVR to be as high as 69%.

The rate of previous surgical interventions was high in our cohort (Table 1). The history of PKP was 58% in the overall group. This is higher than the rate presented by Bové Álvarez et al. [16] (40.8% in the non-trauma group and 17.6% in the trauma group). This is likely due to PKPVR being the standard procedure in our department for patients with simultaneous corneal and retinal pathologies and in need of both PKP and PPV. Although a simultaneous PKPVR might not be necessary in patients without infectious endophthalmitis, it still spares patients from undergoing a second surgical procedure with general anesthesia. Additionally, using an Eckardt keratoprosthesis during the simultaneous PKPVR might also reduce the stress to the corneal graft otherwise occurring during the following PPV. On the other hand, the overall rate of a previous PPV was lower in our cohort (31%) compared to Bové Álvarez et al. [16] (53% in the non-trauma group and 31% in the trauma group).

Similarly, the rate of postoperative interventions was also high in our group (Table 2). A similarly high rate of postoperative interventions was also observed by Bové Álvarez et al. [16] (37% in the non-trauma group and 45% in the trauma group).

Our non-infectious group showed a higher rate of PPV preoperatively compared to the endophthalmitis group. This is likely due to the need of repeat surgical intervention in eyes with complex pathologies after traumatic ocular injury, as well as the occurrence of secondary epiretinal membranes after a previous PPV [23].

The prognosis of PKPVR in the case of IKE showed the greatest variability among the studied indication groups. While the number of enucleations was significantly higher in the IKE-group compared to the non-infectious group (*p* = 0.05), this group also showed a significantly higher potential for significant visual improvement (*p* = 0.04).

Intraoperatively, all IKE patients with significant BCVA improvement had a vital retina without infiltration, ischemia, or hemorrhage, compared to only 3% of patients in the non-significant BCVA improvement group (logMAR ≥ 1.3) (*p* = 0.3). Additionally, all patients with significant BCVA improvement in the IKE-group had no history of PPV compared to 27% of patients who had no significant BCVA improvement (*p* = 0.07). This also underlines the importance of a vital and functional retina to achieve an improvement in BCVA after IKE.

Anatomical success could be reached in 57% of all eyes without enucleation and/or phthisis bulbi (62/93 eyes) without a significant difference between the IKE-group and the non-infectious group. This is close to the anatomical success rate reported by Roters et al. [11] (49%) and somewhat lower than the rate reported by Dong et al. [24] (73%). Anatomical failure occurred due to opaque corneal graft in 32/93 eyes (34%) and/or retinal detachment in 1/93 eyes (1%) and/or poorly regulated IOP in 16/93 eyes (17%). Similarly, a high rate of retinal attachment was reported by Bové Álvarez et al. [16] (90% of eyes in the non-trauma group and 78% of the eyes in the trauma group) and by Roters et al. [12] (92%). On the other hand, a clear corneal graft was more frequently achieved in our cohort compared to Bové Álvarez et al. [16] (66% vs. 50%) and Velez-Montoya et al. [20] (43%), and it was achieved with a similar frequency as Roters et al. obtained [12] (68%). The rate of graft failure was significantly higher in eyes with permanent oil filling compared to those without oil filling. Similar findings were also reported by Yu et al. [25].

Eyes with IKE required repeat PKP significantly more often compared to those without IKE. Further analysis showed that 7/15 eyes required repeat PKP due to the recurrence of the infection, 5/15 due to non-immunological transplant decompensation, 2/15 due to allograft rejection, and 1/15 due to scarring over time. This study is limited due to its retrospective design and the heterogeneity of the non-infectious group. Further studies with a larger cohort are required to further investigate the difference in prognosis between the non-infectious subgroups. The results of other similar studies could also vary depending on the region and its particular pathogens.

In conclusion, PKPVR is a complex surgical procedure which can frequently preserve ocular integrity, avoid phthisis bulbi, and achieve anatomical and, to a lesser extent, functional success in eyes with simultaneous ocular opacity and retinal pathology. PKPVR is an indispensable procedure for eliminating infection in eyes with IKE. Compared to other non-infectious indications for PKPVR, the IKE-group had the highest rate of both enucleation and significant BCVA improvement. The intraoperative presence of retinal hemorrhage, infiltration, and/or ischemia is a significant risk factor for a poor BCVA outcome in patients with IKE. Consequent treatment for ocular surface defects in elderly people is important to avoid these devastating infections. Patients should be made aware of the frequent need for further surgical treatment after PKPVR.

## 5. Video Online

The online version of this article contains a surgical video for “Combined penetrating keratoplasty and pars-plana vitrectomy in a patient with retinal detachment” (https://www.youtube.com/watch?v=GQVsxuC2Gys, accessed on 30 July 2025).

## Figures and Tables

**Table 1:** Demographic characteristics, ocular characteristics, and ocular surgical history.

	Total (129 Eyes)	IKE-Group ^a^ (65 Eyes)	Non-Infection Group (64 Eyes)					*p*-Value
			Total (64 eyes)	Retinal membrane (16 eyes)	Retinal detachment (16 eyes)	IOL luxation (7 eyes)	Trauma (25 eyes)	
Age ^b^ (yrs) Mean ± SD **^a^**	61 ± 22	**68 ± 22**	**54 ± 22**	54 ± 21	56 ± 27	63 ± 14	50 ± 17	**<0.001 ^c^**
Gender (Male–Female)	83:46	**34:31**	**49:15**	11:5	6:1	11:5	21:4	**0.04** ^d^
Lens status Pseudophakic–Phakic–Aphakic	16:78:35	**9:46:10**	**7:32:25**	0:14:2	4:9:3	0:0:7	3:9:13	
**Ocular surgical history**								
Pars plana vitrectomy	40 (31%)	**12 (18%)**	**28 (44%)**	8 (50%)	4 (24%)	1 (14%)	15 (66%)	**0.02** ^d^
Penetrating keratoplasty	75 (58%)	**37 (57%)**	**38 (59%)**	10 (63%)	12 (71%)	5 (71%)	11 (44%)	0.84 ^d^
Glaucoma surgical treatment	22 (17%)	**8 (12%)**	**14 (22%)**	7 (44%)	5 (29%)	1 (14%)	1 (4%)	0.33 ^d^
Amnion membrane transplantation	45 (35%)	**32 (49%)**	**13 (20%)**	4 (25%)	7 (41%)	0	2 (8%)	**<0.001** ^d^

^a^ IKE: infectious keratitis with endophthalmitis; SD: standard deviation. ^b^ Age calculated at date of combined penetrating keratoplasty and pars plana vitrectomy. ^c^ Comparison between IKE-group and non-infection group using Student’s *t*-test. Bold *p*-value: significant *p*-value. ^d^ Comparison between IKE-group and non-infection group using Chi-square test.

**Table 2:** Functional and anatomical results and postoperative surgical procedures.

	Total (107 Eyes)	IKE-Group ^a^ (50 Eyes)	Non-Infection Group (57 Eyes)					*p*-Value
			Total (57 eyes)	Epiretinal membrane (15 eyes)	Retinal detachment (13 eyes)	IOL luxation (6 eyes)	Trauma (23 eyes)	
BCVA ^a^ at baseline visit	2.3 ± 0.5	**2.3 ± 0.3**	**2.2 ± 0.5**	2.0 ± 0.5	2.4 ± 0.3	1.8 ± 0.8	2.3 ± 0.5	0.16 ^c^
BCVA at last visit	2.0 ± 0.7	**2.0 ± 0.7**	**2.1 ± 0.6**	1.7 ± 0.6	2.3 ± 0.5	1.8 ± 0.5	2.2 ± 0.6	0.3 ^c^
*p*-value ^b^	**<0.001**	**<0.001**	**0.02**	**0.01**	0.6	1	0.16	
Significant BCVA improvement ^a^	12 (13%)	**9 (19%)**	**3 (6%)**	2 (15%)	0	1 (20%)	0	**0.04** ^d^
Postoperative BCVA < 1.3	15 (14%)	**9 (19%)**	**6 (11%)**	4 (27%)	0	1 (16%)	1 (4%)	0.26 ^d^
Change in BCVA after PKPVR ^a^: Better–same–worse	53:34:20	**30:11:9**	**23:23:11**	7:7:1	5.3:5	3:2:1	8:11:4	
**Anatomical results**								
Enucleation	9	**7**	**2**	0	1	0	1	**0.05** ^d^
Phthisis bulbi	5	**2**	**3**	0	1	0	2	0.67 ^d^
Clear corneal graft	61/93 (66%)	**30/41 (73%)**	**31/52 (60%)**	11/15 (73%)	6/11 (55%)	4/6 (66%)	10/20 (50%)	0.17 ^d^
Retinal attachment	92/93 (99%)	**40/41 (98%)**	**52/52 (100%)**	15/15 (100%)	11/11 (100%)	6/6 (100%)	20/20 (100%)	
Eyes filled with oil at last visit	13/93 (14%)	**4/41 (9%)**	**9/52 (17%)**	0	0	5/15 (33%)	4/20 (20%)	
Normal IOP at last visit	77/93 (83%)	**35/41 (85%)**	**42/52 (81%)**	12/15 (80%)	9/11 (82%)	6/6 (100%)	15/20 (75%)	
Anatomical success	53/93 (57%)	**27/41 (66%)**	**26/52 (50%)**	9/15 (60%)	5/11 (45%)	4/6 (66%)	8/20 (40%)	0.12 ^d^
**Postoperative surgical procedures**								
Pars plana vitrectomy	32/107 (30%)	**18 (36%)**	**28 (49%)**	3 (20%)	4 (31%)	1 (17%)	6 (26%)	0.17 ^d^
Penetrating keratoplasty	18 (17%)	**15 (30%)**	**3 (5%)**	1 (7%)	0	0	2 (9%)	**<0.001** ^d^
Glaucoma surgical treatment	18 (17%)	**3 (6%)**	**15 (26%)**	7 (47%)	1 (8%)	2 (33%)	5 (22%)	**0.005** ^d^
Amnion membrane transplantation	30 (28%)	**19 (38%)**	**11 (19%)**	3 (20%)	3 (23%)	0	5 (22%)	**0.03** ^d^
PKPVR	16 (15%)	**11 (22%)**	**6 (11%)**	3 (20%)	0	0	3 (13%)	0.1 ^d^

^a^ IKE: infectious keratitis with endophthalmitis; BCVA: best corrected visual acuity; PKPVR: combined penetrating keratoplasty and vitreoretinal surgery; Significant BCVA improvement: defined as improvement in BCVA from ≥1.3 logMAR preoperatively to BCVA < 1.3 postoperatively. ^b^ Comparison between BCVA at baseline visit and at last visit using paired Student’s *t*-test. Bold *p*-value: significant *p*-value. ^c^ Comparison between IKE-group and non-infection group using Student’s *t*-test. ^d^ Comparison between IKE-group and non-infection group using Chi-square test.

**Table 3:** Comparison of eyes with and without significant visual improvement ^a^ in patients with IKE ^a^.

	Significant BCVA Improvement (9 Eyes)	Non-Significant BCVA Improvement (41 Eyes)	*p*-Value
**Preoperative surgical procedures**			
Pars plana vitrectomy	0	11 (27%)	0.07 ^b^
Penetrating keratoplasty	5 (56%)	28 (68%)	0.49 ^b^
Glaucoma surgical treatment	0	8 (20%)	0.14 ^b^
Amnion membrane transplantation	4 (44%)	26 (63%)	0.09
**Intraoperative retinal pathologies (retinal infiltration, bleeding, and/or ischemia)**	0	15 (37%)	**0.03** ^b^
**Postoperative surgical procedures**			
Pars plana vitrectomy	3 (30%)	15 (38%)	0.16 ^b^
Penetrating keratoplasty	1 (11%)	14 (34%)	0.17 ^b^
Glaucoma surgical treatment	1 (11%)	2 (5%)	0.4 ^b^
Amnion membrane transplantation	4 (44%)	15 (37%)	0.64 ^b^
PKPVR ^a^	3 (33%)	8 (19%)	0.37 ^b^

^a^ IKE: infectious keratitis with endophthalmitis; PKPVR: combined penetrating keratoplasty and vitreoretinal surgery. Significant BCVA improvement: defined as improvement in BCVA from ≥1.3 logMAR preoperatively to BCVA < 1.3 postoperatively. ^b^ Comparison between IKE-group and non-infection group using Chi-square test. Bold *p*-value: significant *p*-value.

## Data Availability

Data are available from the corresponding author upon request.

## References

[1] Frisina R., Besozzi G., Gius I., Greggio A., De Salvo G., Meduri A. (2022). Pole to Pole Surgery in Ocular Trauma: Standardizing Surgical Steps. Ophthalmol. Ther..

[2] Ikeda T. (2001). Pars plana vitrectomy combined with penetrating keratoplasty. Semin. Ophthalmol..

[3] Mayalı H., Kayıkçıoğlu Ö., Altınışık M., Bıçak F., Kurt E. (2019). Clinical results in patients with combined penetrating keratoplasty and vitreoretinal surgery using landers wide-field temporary keratoprosthesis. Turk. J. Ophthalmol..

[4] Durand M.L. (2013). Endophthalmitis. Clin. Microbiol. Infect..

[5] Lu X., Ng D.S.C., Zheng K., Peng K., Jin C., Xia H., Chen W., Chen H. (2016). Risk factors for endophthalmitis requiring evisceration or enucleation. Sci. Rep..

[6] Alfaro Rangel R., Szentmáry N., Lepper S., Milioti G., Daas L., Langenbucher A., Seitz B. (2022). Large-diameter penetrating keratoplasties are mostly due to very severe infectious keratitis and cannot always prevent secondary enucleation. Klin. Monatsblätter Für Augenheilkd..

[7] McGwin G., Xie A., Owsley C. (2005). Rate of Eye Injury in the United States. Arch. Ophthalmol..

[8] Gelston C.D., Deitz G.A. (2020). Eye Emergencies. Am. Fam. Physician.

[9] Schulze-Bonsel K., Feltgen N., Burau H., Hansen L., Bach M. (2007). Author Response: Numerical Imputation for Low Vision States Numerical Imputation for Low Vision States. IOVS Invest. Ophthalmol. Vis. Sci..

[10] Schulze-Bonsel K., Feltgen N., Burau H., Hansen L., Bach M. (2006). Visual Acuities “Hand Motion” and “Counting Fingers” Can Be Quantified with the Freiburg Visual Acuity Test. Investig. Ophthalmol. Vis. Sci..

[11] Finger R.P., Fimmers R., Holz F.G., Scholl H.P.N. (2011). Incidence of Blindness and Severe Visual Impairment in Germany: Projections for 2030. Investig. Ophthalmol. Vis. Sci..

[12] Roters S., Hamzei P., Szurman P., Hermes S., Thumann G., Bartz-Schmidt K., Kirchhof B. (2003). Combined penetrating keratoplasty and vitreoretinal surgery with silicone oil: A 1-year follow-up. Graefes Arch. Clin. Exp. Ophthalmol..

[13] Fiorentzis M., Morinello E., Viestenz A., Zuche H., Seitz B., Viestenz A. (2017). Muscle relaxants as a risk factor for vis-à-tergo during penetrating keratoplasty: A prospective interventional study. Adv. Ther..

[14] Garcia-Valenzuela E., Blair N.P., Shapiro M.J., Gieser J.P., Resnick K.I., Solomon M.J., Sugar J. (1999). Outcome of vitreoretinal surgery and penetrating keratoplasty using temporary keratoprosthesis. Retina.

[15] Sheu S.J. (2017). Endophthalmitis. Korean J. Ophthalmol..

[16] Bové Álvarez M., Arumí C.G., Distéfano L., Güell J.L., Gris Ó., Mateo C., Corcóstegui B., García-Arumí J. (2019). Comparative study of penetrating keratoplasty and vitreoretinal surgery with Eckardt temporary keratoprosthesis in ocular trauma versus non-trauma patients. Graefe’s Arch. Clin. Exp. Ophthalmol..

[17] Zapata Cuevas M.A., García de Oteyza G., Alvarado-Villacorta R., Hernández L.M.Q., Ordoñez-Ranz G., de Wit Carter G., García-Albisua A.M. (2023). Pars plana vitrectomy and therapeutic keratoplasty in endophthalmitis and infectious keratitis. Eur. J. Ophthalmol..

[18] Cooney T., Kinast R., Juratli L., Pedreira P.N., Saxe S., Musch D.C., Mian S.I. (2023). Combined Penetrating Keratoplasty and Vitreoretinal Surgery With Temporary Keratoprosthesis. Cornea Open.

[19] Dave A., Acharaya M., Agarwal M., Dave P.A., Singh M., Mathur U. (2019). Outcomes of combined keratoplasty and pars plana vitrectomy for endophthalmitis with compromised corneal clarity. Clin. Exp. Ophthalmol..

[20] Velez-Montoya R., Rivera-Cortes M.A., Ledesma-Gil G., Carranza-Casas M., Martinez J.D., Levine H., Yanuzzi N.A., Amescua G., Ahmed I., Beatson B. (2023). Combined Therapeutic Penetrating Keratoplasty and Pars Plana Vitrectomy for the Treatment of Infectious Keratitis Endophthalmitis: Mexican Endophthalmitis Study Group Protocol 4. Cornea.

[21] Saad S., Abdelmassih Y., Saad R., Guindolet D., el Khoury S., Doan S., Cochereau I., Gabison E.E. (2020). Neurotrophic keratitis: Frequency, etiologies, clinical management and outcomes. Ocul. Surf..

[22] Pelletier J., Koyfman A., Long B. (2023). High risk and low prevalence diseases: Open globe injury. Am. J. Emerg. Med..

[23] Wu R.H., Xu M.N., Lin K., Ren M.-X., Wen H., Feng K.-M., Zhou H.-J., Moonasar N., Lin Z. (2022). Inner limiting membrane peeling prevents secondary epiretinal membrane after vitrectomy for proliferative diabetic retinopathy. Int. J. Ophthalmol..

[24] Dong X., Wang W., Xie L., Chiu A.M.C. (2006). Long-term outcome of combined penetrating keratoplasty and vitreoretinal surgery using temporary keratoprosthesis. Eye.

[25] Yu J., Shalaby W.S., Shiuey E.J., Rapuano C.J., Yonekawa Y., Hammersmith K.M., Nagra P.K., Syed Z.A. (2023). Graft Outcomes After Temporary Keratoprosthesis in Combined Penetrating Keratoplasty and Vitreoretinal Surgery. Cornea.

